# Synthesis, Characterization, and Biologic Activity of New Acyl Hydrazides and 1,3,4-Oxadiazole Derivatives

**DOI:** 10.3390/molecules25143308

**Published:** 2020-07-21

**Authors:** Irina Zarafu, Lilia Matei, Coralia Bleotu, Petre Ionita, Arnaud Tatibouët, Anca Păun, Ioana Nicolau, Anamaria Hanganu, Carmen Limban, Diana Camelia Nuta, Roxana Maria Nemeș, Carmen Cristina Diaconu, Cristiana Radulescu

**Affiliations:** 1 Faculty of Chemistry, University of Bucharest, 050663 Bucharest, Romania; petre.ionita@chimie.unibuc.ro (P.I.); anca.paun@chimie.unibuc.ro (A.P.); ioana.nicolau@chimie.unibuc.ro (I.N.); 2 “Stefan S Nicolau” Institute of Virology, Romanian Academy, 030304 Bucharest, Romania; lilia.matei@virology.ro (L.M.); coralia.bleotu@virology.ro (C.B.); carmen.diaconu@virology.ro (C.C.D.); 3 Research Institute of the University of Bucharest (ICUB), Life, Environmental and Earth Sciences Division, University of Bucharest, 060023 Bucharest, Romania; anamaria.hanganu@unibuc.ro; 4 Institute of Organic and Analytical Chemistry ICOA-UMR7311, University of Orleans, 45067 Orleans, France; arnaud.tatibouet@univ-orleans.fr; 5 Institute of Organic Chemistry “C.D. Nenitescu” of the Romanian Academy, 060023 Bucharest, Romania; 6 Faculty of Medicine, “Carol Davila” University of Medicine and Pharmacy, 020021 Bucharest, Romania; carmen.limban@umfcd.ro (C.L.); diana.nuta@umfcd.ro (D.C.N.); 7 National Institute of Pneumology Marius Nasta, 050152 Bucharest, Romania; roxanamarianemes@gmail.com; 8 Faculty of Sciences and Arts, “Valahia” University of Targoviste, 130004 Targoviste, Romania; 9 Institute of Multidisciplinary Research for Science and Technology, Valahia University of Targoviste, 13004 Targoviste, Romania

**Keywords:** isoniazid, oxadiazole, synthesis, antibacterial activity, apoptosis, cell cycle, drug metabolism genes expression

## Abstract

Starting from isoniazid and carboxylic acids as precursors, thirteen new hydrazides and 1,3,4-oxadiazoles of 2-(4-substituted-phenoxymethyl)-benzoic acids were synthesized and characterized by appropriate means. Their biological properties were evaluated in terms of apoptosis, cell cycle blocking, and drug metabolism gene expression on HCT-8 and HT-29 cell lines. In vitro antimicrobial tests were performed by the microplate Alamar Blue assay for the anti-mycobacterial activities and an adapted agar disk diffusion technique for other non-tubercular bacterial strains. The best antibacterial activity (anti-*Mycobacterium tuberculosis* effects) was proved by **9**. Compounds **7**, **8**, and **9** determined blocking of G1 phase. Compound **7** proved to be toxic, inducing apoptosis in 54% of cells after 72 h, an effect that can be predicted by the increased expression of mRNA caspases 3 and 7 after 24 h. The influence of compounds on gene expression of enzymes implicated in drug metabolism indicates that synthesized compounds could be metabolized via other pathways than NAT2, spanning adverse effects of isoniazid. Compound **9** had the best antibacterial activity, being used as a disinfectant agent. Compounds **7**, **8**, and **9**, seemed to have antitumor potential. Further studies on the action mechanism of these compounds on the cell cycle may bring new information regarding their biological activity.

## 1. Introduction

Hydrazides represent a class of organic compounds containing a nitrogen–nitrogen covalent bond with at least one acyl substituent [1]. They are used as starting materials for the synthesis of surfactants and of various heterocycles as of 1,2,4-triazoles [2], 1,3,4-thiadiazoles [3], or 1,3,4-oxadiazoles [3,4] with pharmacological activity [5]. Hydrazides are well known for their antitumor [6], anti-inflammatory [7], analgesic [8], antibacterial [7,9], and antiviral [10] activities, while 1,3,4-oxadiazoles are known for antibacterial, antifungal, and anti-inflammatory activities [11,12].

Is well known that isoniazid (INH) is used as valuable drug for anti-tuberculosis treatments mostly against the active metabolize and multiply of *Mycobacterium tuberculosis* bacteria [13,14], but less effective than dormant ones [15]. The hydrazide of isonicotinic acid is considered a prodrug capable of crossing the *M. tuberculosis* cell wall through passive diffusion [16]. After reaching the inside of the cell [17], KatG (mycobacterial catalase-oxidase) is activating it. The active forms of INH shown different effects on the cell wall mycolic acids synthesis [18], on nucleic acids replication/transcription [19], and on bacterial respiratory metabolism, as well [20]. All this time, it never been completely discovered the complex mechanism of action of isoniazid as prodrug [21,22].

In terms of toxicity effect over the human body, the INH behavior is different for each patient. The isoniazid is degradaded mainly by acetylation with N-acetyltransferase (NAT), which conduct to N1-acetyl-N2-isonicotinylhydrazine (acetylisoniazid) metabolite, and further, by metabolization process, result the mono-acetylhydrazine and the nontoxic di-acetylhydrazine [23,24,25]. The polymorphisms of genes that codify NATs, also influence the metabolism of INH. On the other hand, different degrees of activity for NATs and an individual acetylation profile are determined by the presence of various polymorphisms [26]. On this basis, the humans may be classified in three classes such as slow, intermediate, and fast acetylators [27]. Therefore, a fast acetylation of INH lead to a lowering concentration of active drug [14], being responsible for applied therapy effectiveness, mostly to some patients which required single weekly doses [28]. On the other hand, for slow acetylators the risk for hepatotoxic effects occurrence is possible due to high concentrations of isoniazid metabolites [24,29,30]. Literature [22,31,32] revealed that the any change added to isoniazid molecule such as the inclusion of a functional group to the hydrazine structure could block the acetylation of the drug by NATs, therefore influencing its activity and toxicity.

It had been previously shown that isoniazid derivatives exhibited a good anti-mycobacterial activity, some of them being more active than isoniazid or other anti-tubercular drugs used in the current therapy regimens [33,34,35,36,37,38]. Various studies have postulated that the majority of isoniazid derivatives are activated before reaching the intracellular medium of *M. tuberculosis* [39]. On the other hand, based on QSAR study [40], the Ventura group presumed that isoniazid derivatives activation involve the formation of electrophilic intermediate species (hydrazyl radical or ion) that are transformed in acyl radicals which can couple with NADH/NAD^+^, the formed adducts inhibiting enoyl-acyl carrier protein reductase (InhA) activity [41].

To the best of our knowledge, the synthesis of new isoniazid derivatives based clinical drugs is important for the therapeutic field. These observations encouraged the authors to continue the previous research (i.e., synthesis, characterization, and pharmacological evaluation of new heterocyclic compounds), where hydrazides/indazoles/oxadiazoles linked with other heterocycles proved as effective antimicrobial agents [42,43,44,45,46,47,48]. The purpose of current study was to characterize the new synthesized isoniazid derivatives and to preliminary evaluate their biological activity to identify their usefulness as drugs in medical field.

## 2. Results and Discussion

### 2.1. Synthesis and Structural Characterization

The benzoic acid hydrazides were prepared following the method described in the general experimental procedure. The carboxylic acids were obtained by a method reported earlier by Limban group [49], by refluxing the phthalide with potassium p-substituted phenolates in xylene. The obtained corresponding acid was treated with thionyl chloride in 1,2-dichloroethane under reflux. The crude acid chloride thus obtained was added to isoniazid dissolved in dichloromethane and the reaction mixture was stirred at room temperature. Product formation was monitored by thin-layer chromatography on silica gel, and the obtained products were separated and purified on chromatographic columns. In the case of 2-(4-methyl/methoxy/ethyl-phenoxymethyl)-benzoic acid derivatives, the presence of two new compounds in each reaction was observed.

The newly synthesized *N*,*N*’-diacylhydrazines (**1**–**6**) were diluted in toluene and treated with phosphoryl chloride. The reaction mixture was refluxed for 6 h and then stirred overnight at room temperature. Product formation was monitored by thin layer chromatography on silica gel. Toluene and residual phosphoryl chloride were removed under reduced pressure and the reaction products were taken up in dichloromethane and washed with 5% NaOH solution and with saturated aqueous NaCl solution. The organic layer was dried over anhydrous sodium sulfate and the solvent distilled under reduced pressure. The newly synthesized 1,3,4-oxadiazoles were recrystallized using ethanol.

**Figure 1:**
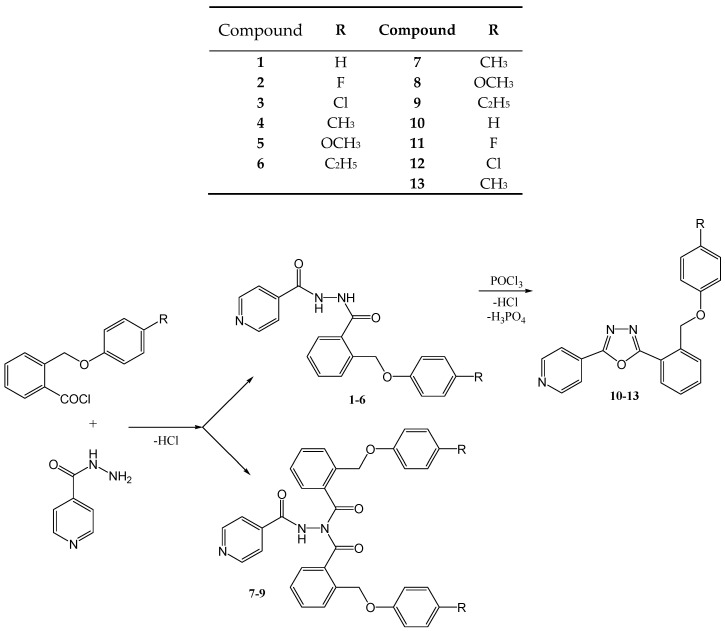
Synthesis of the new isoniazid derivatives.

The synthesis reactions of new compounds are presented in Figure 1.

The synthesis yields (attempts to optimize were not made), the melting points, and the *R_f_* values of the new compounds obtained are presented in Table 1.

The chemical structure of the newly synthesized compounds was confirmed by ^1^H-NMR, ^13^C-NMR, IR, and by elemental analysis. The ^1^H-NMR spectra of compounds **1**–**6** showed two singlets at 10.11–11.20 ppm and 9.72–10.55 ppm, which represent the two hydrazide protons (NH-NH, deuterable), while the ^1^H-NMR spectra of compounds **7**–**9** showed only one singlet at 8.53–11.97 ppm, corresponding to the NH–N proton. This indicates that compounds **1**–**6** are *N,N’*-disubstituted, while compounds **7**–**9** are *N*,*N,N’*-trisubstituted.

FTIR spectra showed the C=O peak at 1690–1669 cm^−1^ and the C–N peak at 1609–1592 cm^−1^, and, also, in the case of compounds **1**–**9**, the N–H stretching vibration at 3271–3191 cm^−1^. The stretching vibrations of the aromatic rings (C=C–C) are situated at 1608–1443 cm^−1^. For all compounds it can distinguish the 1,2- and 1,4-disubstitution at 748–735 cm^−1^ and 841–811 cm^−1^, respectively. The complete interpretation of ^1^H-, ^13^C-NMR and IR spectra and the elemental analyses of new compounds are presented in the experimental part.

All compounds were subjected to MS analysis, the signals of molecular ions [M + H] corresponding to the calculated molecular weight and structure of these compounds.

### 2.2. Biological Evaluation

#### 2.2.1. Antimicrobial Activity

Taking into account that isoniazid derivatives are known for their anti-tubercular activity [30,32,36], the newly synthesized derivatives of isoniazid were tested for their in vitro activity against a clinical strain of *M. tuberculosis*. The tested compounds were quantitatively assayed using serial binary dilutions ranging from 25 μg/mL to 0.012 μg/mL. None of the new synthesized compounds proved to have anti-tubercular activity, apart from compound **9** that inhibited the growth of *M. tuberculosis* at a concentration of 6.25 μg/mL (Table 2).

The minimal inhibitory concentration (MIC) of the compound **9** was moderate, which is in the range of those reported for other isoniazid derivatives obtained by combination with acid chlorides [33,34,35,36,37,38]. A very active derivative against *M. tuberculosis* is 1-isonicotinyl-2-nonanoyl hydrazine, superior to isoniazid with a very low minimal inhibitory concentration value of 0.025 µg/mL [38]. Other compounds from this class exhibited MIC values ranging between 0.39 and 71.6 µg/mL [33,34,35,36,37]. Regarding the isoniazid-derived 1,3,4-oxadiazoles reported in literature, few of them proved anti-tuberculosis activity [50].

In order to establish if these new synthesized compounds have antibacterial activity against other microbial species and to determine the spectrum of their activity, the novel derivatives were tested on other, non-tuberculosis, bacterial strains isolated from clinical specimens, both Gram-positive (e.g., *Staphylococcus hominis*, *Staphylococcus aureus*, and coagulase-negative staphylococci) and Gram-negative (e.g., *Escherichia coli*, *Klebsiella pneumoniae*, *Proteus mirabilis*, *Citrobacter koseri*, *Morganella morganni*, *Acinetobacter baumanii*, and *Pseudomonas aeruginosa*), using an adapted diffusion technique. The most active derivative proved to be compound **9**, which inhibited the growth of the majority of tested strains, followed by the compounds **6** and **7**. The less active derivatives were compounds **1**, **3**, and **10**–**13**. On the other hand, the *N,N,N’*-triacylhydrazines **7**–**9** exhibited a better antibacterial activity than their corresponding *N,N’*-diacylhydrazines **4**–**6**, as well as the rest of tested compounds (Table 3). According to previously reported data compounds with similar structures proved inhibitory activity on the growth of *Staphylococcus aureus*, *Enterococcus faecalis*, *Pseudomonas aeruginosa*, and *Escherichia coli* [33,34,35,36,37].

#### 2.2.2. The Influence of the New Isoniazid Derivatives on the Expression Levels of Some Genes Implicated in Drug Metabolism

**Figure 2:**
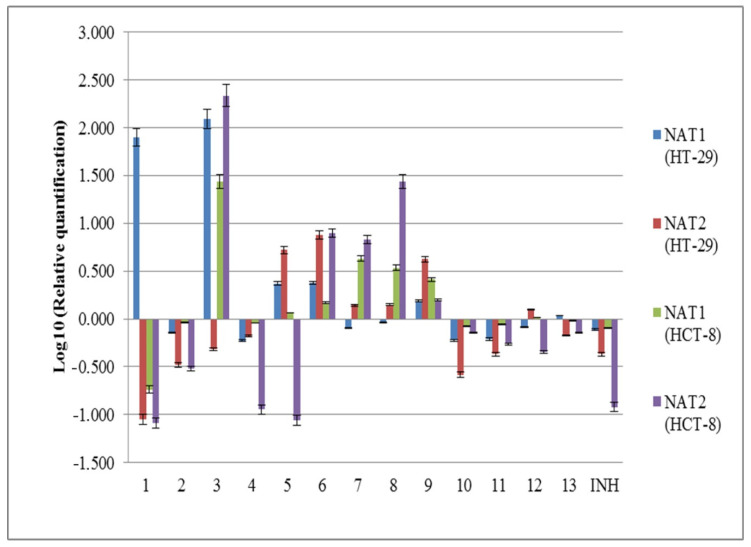
The influence of the new isoniazid derivatives **1**–**13** on the expression of *NAT1* and *NAT2* genes in HT-29 and HCT-8 cell lines.

The main metabolic pathway of isoniazid and similar compounds in human body involves its N-acetylation and the increased expression of *NAT1* and *NAT2* genes, which is suggested to be associated with a better rate of metabolization. In this context, the influence of newly synthesized substances on *NAT1* and *NAT2* expression in eukaryotic cells was evaluated in two cell lines—HCT-8 and HT-29—at a concentration of 50 µg/mL. As we previously reported, the HT-29 cell line presents NAT2*5 genotype, while the HCT-8 cell line presents the NAT2*6 genotype [51]. Isoniazid decreased the expression of *NAT1* and *NAT2* in both cell lines, as well as compounds **2**, **4**, **10**, and **11**. *NAT1* and *NAT2* gene expressions were increased by compounds **6** and **9** in both HT-29 and HCT-8 cell lines. Compounds **3** and **5** increased the gene expression of *NAT1* in both used cell lines, compound **1** increased *NAT1* expression only in HT-29, and compounds **7** and **8** increased it only in the HCT-8 cell line. Regarding *NAT2* gene expression, it was increased by compounds **7** and **8** in both tested cell lines, by compounds **5** and **12** in HT-29 cell line, and by compound **3** in HCT-8 cells. Generally, the *NAT2* gene expression was more influenced by tested compounds in HCT-8 cell line, than in HT-29 cell line, with the exception of compounds **9**–**11** (Figure 2). Compounds **6**–**9** could be used to potentiate isoniazid metabolism in slow acetylators, and further studies should be done in this direction.

**Figure 3:**
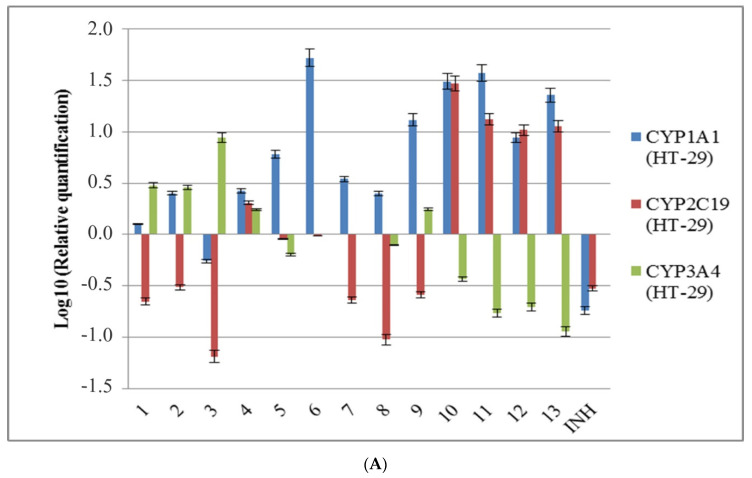

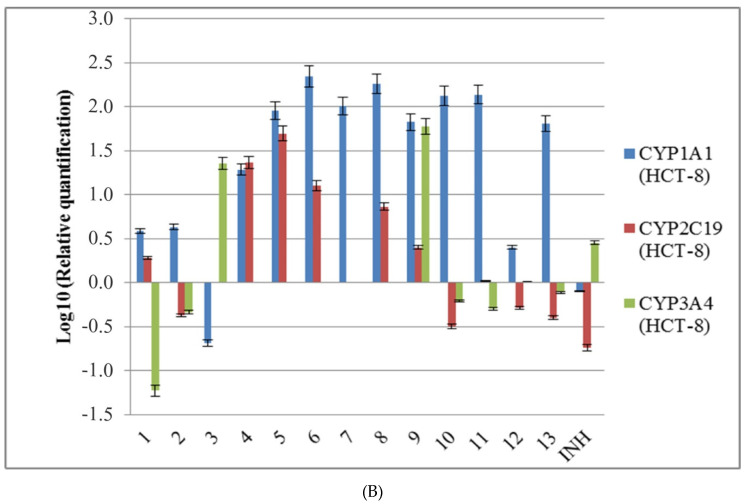
The influence of the new isoniazid derivatives **1**–**13** on the expression of CYP1A1, CYP2C19, and CYP3A4 genes in the eukaryotic cells; (**A**) HT-29 cells; (**B**) HCT-8 cells

In order to identify how new synthesized compounds may be degraded and eliminated from the body, the expression of some CYP450 isoenzymes, involved in the metabolism of pharmacological substances, was evaluated in HCT-8 and HT-29 cell lines (Figure 3). The obtained results shown that isoniazid decreased the expression of *CYP1A1* and *CYP2C19* genes in both cell lines, but increased the expression of *CYP3A4* in HCT-8 cells. All tested compounds increased *CYP1A1* expression in both cell lines, except for compound **3**. Regarding the expression of *CYP2C19*, it was increased by compound **4** in both used cell lines; by compounds **1**, **5**, **6**, **8**, and **9** in HCT-8 cells; and by new synthesized 1,3,4-oxadiazoles **10**, **11**, **12**, and **13** in HT-29 cells. *CYP3A4* gene expression was increased by compounds **3** and **9** in HT-29 and in HCT-8 cells, and by compounds **1, 2,** and **4** only in HT-29 cells.

Isoniazid inhibits *CYP2C19* and *CYP3A4* activities and mechanistically inactivates *CYP1A2*, *CYP2A6*, *CYP2C19*, and *CYP3A4* in human liver microsomes [52,53]. El Sayed’s group analyzed the influence of some isoniazid derivatives on the expression of mRNA and activity of enzymes implicated in drug metabolism in mice [54]. Although the analyzed compounds determined some changes in mRNA expressions, their influence on enzymes activity was minor [54]. The increased expression levels of *CYP1A1* induced by the new synthesized isoniazid derivatives could be explained by the induction of their metabolism. CYP450 enzymes are implicated in phase I of xenobiotic and drug metabolism, and *CYP1A1* catalyzes the hydroxylation and other oxidative transformations of aromatic substances [53,55]. Regarding different profiles of *CYP2C19* expression in the two cell lines used, this could account for the existence of a polymorphic locus.

#### 2.2.3. Cytotoxicity and Effects on Cell Cycle

The obtained compounds have also been tested to establish their cytotoxicity and the apoptotic effect on HT-29 cell line. The effect of the treatment with 50 μg/mL of substances for 24, 48, and 72 h was evaluated by flow cytometry using FITC-labeled annexin-V and propidium iodide staining that discriminate between apoptotic and intact cells. The most toxic compound proved to be compound **7**, followed by compounds **8** and **9**. The treatment of HT-29 cells with 50 µg/mL of compound **7** has no toxic effect after 24 h, but after 48 and 72 h it induced necrosis in 28.6% and 54%, respectively, of the eukaryotic cells (Table 4).

Kumar’s group evaluated a series of *N’*-(*E*)-(substituted-benzylidene)-isonicotinohydrazide derivatives regarding their anticancer activity against several types of tumors and determined that isonicotinoyl hydrazides display moderate to potent anticancer activity [56]. Later, Rodrigues et al. evaluated thirty-two *N’*-[(*E*)-(disubstituted-phenyl)-methylidene]-isonicotino-hydrazides for their activity against four human cancer cell lines and established that the biological activity of this compounds is determined by the number, the positions, and the types of substituents attached to the aromatic ring. The best results were proved by disubstituted derivatives, and the presence of hydroxyl groups on the benzene ring plays an important role in the anticancer activity of this series, especially when it is located in *o*-position. Hydroxyl groups located in *o*-position in hydrazone systems are good ligands for metals, the action mechanism of this class could possibly be based on the formation of complexes that are likely to inactivate enzymes involved in abnormal cell division [57].

In current case, the most cytotoxic activity had been proved by a compound containing a methyl group in *p*-position of a benzene ring (compound **7**). This effect could be probably explained by its metabolism, the tolyl moiety being oxidized to the corresponding alcohol and carboxylic acid and to *o*-cresyl [58], the presence of hydroxyl groups on the benzene ring in *o*-position playing an important role in the anticancer activity [57]. The metabolic pathway of compound **9** possibly involves the oxidation of ethyl group resulting the corresponding alcohols and acids, which are less toxic [59]. In the case of compound **8**, the major metabolic pathway could involve O-demethylation of this compound, similar to other compounds containing *p*-methoxyphenyl moiety [60], with isoniazid derivatives containing a hydroxyl moiety in the *p*-position of a benzene ring being less toxic [57].

**Figure 4:**
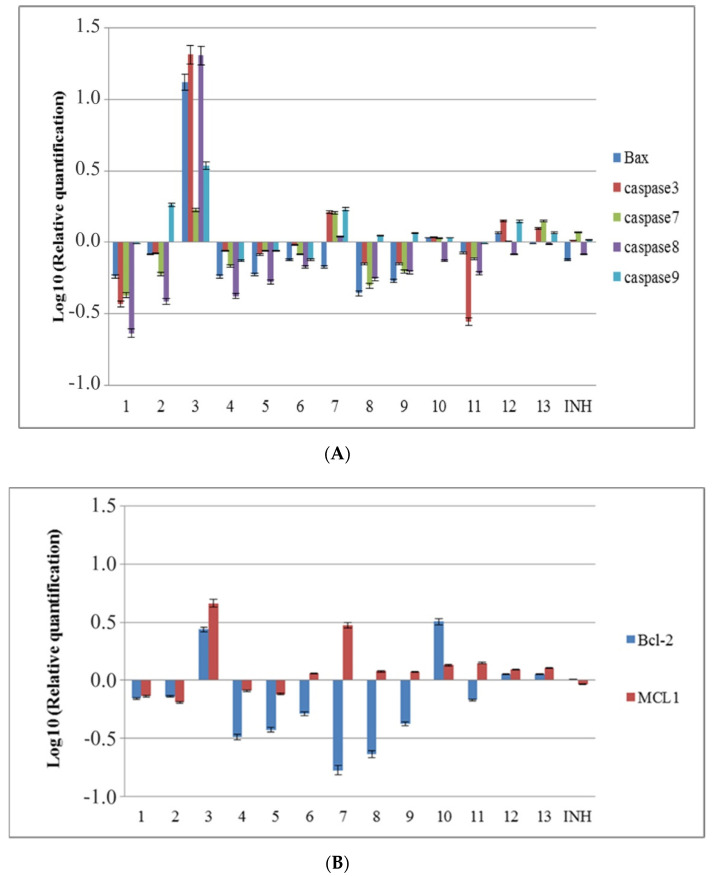
The influence of the new isoniazid derivatives on the expression of genes implicated in apoptosis induction in HCT-8 cells; (**A**) pro-apoptotic genes; (**B**) anti-apoptotic genes.

The cytotoxicity of the newly synthesized compounds was also studied at the molecular level by analyzing the expression of genes implicated in apoptosis. From all tested substances, compounds **3** and **7** determined an increase in the expression levels of *caspases 3*, *7*, *8*, and *9* genes. Compounds **10** and **13** slightly increased the gene expression of *caspases 3*, *7*, and *9*, and compound **12** increased *caspase 3* and *9* gene expression, while compounds **2**, **8**, and **9** increased only the expression of *caspase 9*. Regarding the proapoptotic *Bax* (Bcl-2-associated X protein) and antiapoptotic *Bcl-2* (B-cell lymphoma 2), their gene expression was increased by compounds **2**, **10**, and **12**. Compound **13** increased *Bcl-2* and decreased *Bax* gene expressions, while other tested compounds decreased both genes expressions. Still, the *Bax*/*Bcl-2* ratio was positive accounting for an induction of apoptotic program. Regarding *MCL1* (Myeloid Cell Leukemia Sequence 1), compounds **1**, **2**, **4**, and **5** decreased expression of this gene, while the other compounds increased it (Figure 4).

The activation of the effector caspase 3 can be induced by the extrinsic pathway via tumor necrosis factor (TNF) family receptors (e.g., Fas), FADD (Fas-activated death domain protein), and caspase 8, or by the intrinsic pathway via the mitochondrial release of cytochrome c and Apaf-1–mediated processing of caspase 9 [61]. In the case of compound **7**, the increased expression of *caspase 9* could be associated with activation of intrinsic apoptotic pathway. Although the phenotypic changes induced by the treatment of compound **7** with 50 µg/mL for 24 h are minor, the increased expression of mRNA *caspase 3* and *7* predicts the apoptotic phenomena occurring at 48 and 72 h. In the case of compounds **3**, **12**, and **13**, although all evaluated caspases and *Bax* gene expressions were increased, the expression of antiapoptotic *Bcl-2* and *MCL1* genes was also high, and no apoptotic effect was observed at 48 or 72 h.

**Figure 5:**
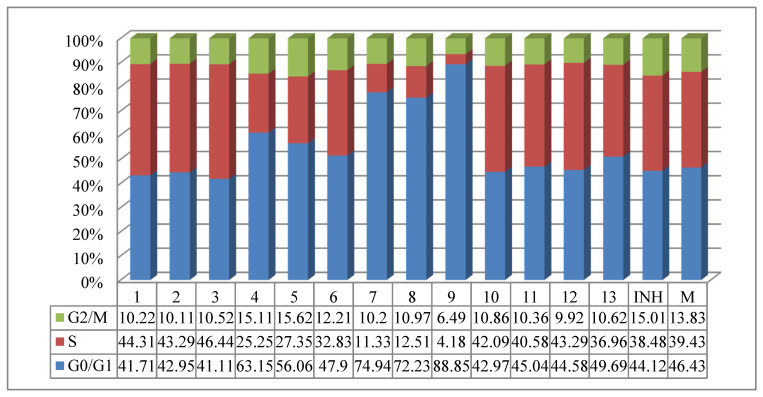
The influence of the new isoniazid derivatives on cell cycle in eukaryotic cells after a 24 h treatment.

**Figure 6:**
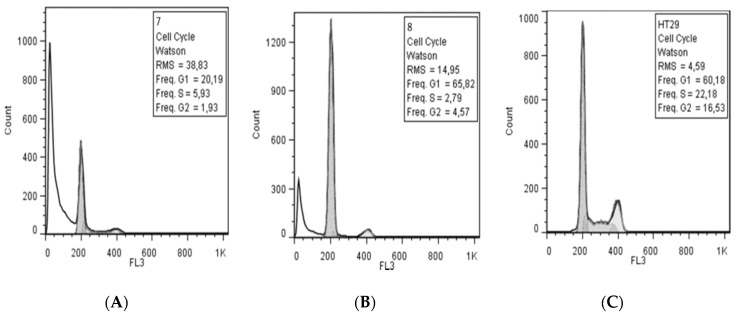
The influence of compounds **7** and **8** on the cell cycle in eukaryotic cells after a 48-h treatment: (**A**) compound 7; (**B**) compound 8; (**C**) control cells (HT 29).

The influence of the new compounds on the cell cycle was determined on HCT-8 cells treated for 24 h with 50 μg/mL of the tested compounds. Compounds **4**–**9** and **13**, containing methyl, ethyl, or methoxy moieties, increased the G0/G1 phase and decreased the S phase, while the other compounds decreased the G0/G1 phase and increased the S phase. Compounds **4**–**6** slightly increased the G2/M phase, while the rest of compounds decreased this phase. *N,N,N’*-Triacylhydrazines **7**–**9** exhibited a stronger influence on the cell cycle than the other tested compounds (Figure 5). The evaluation of the treatment at 48 h showed a decrease of S and G2/M phases in the case of *N,N,N’*-triacylhydrazines **7** and **8**, and in addition, the appearance of the subG0 peak associated with apoptosis (Figure 6).

**Figure 7:**
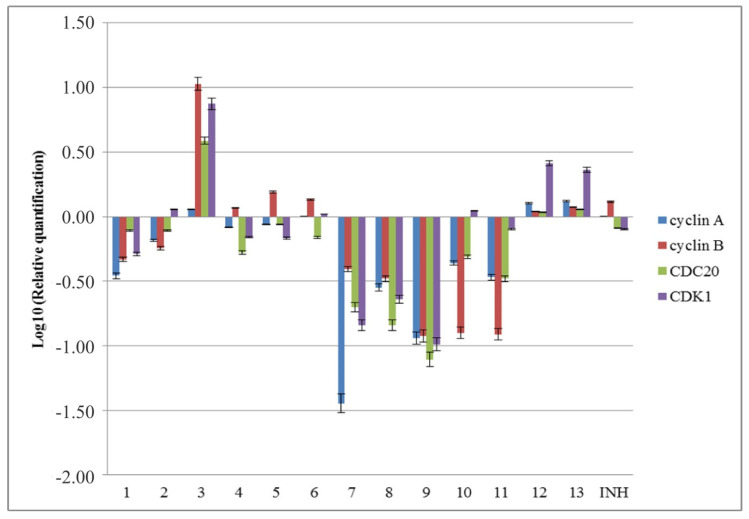
The influence of the new isoniazid derivatives **1**–**13** on the expression of *cyclin A*, *cyclin B*, *CDK1*, and *CDC20* genes in the eukaryotic cells.

At molecular level, the cell cycle is regulated by cyclin-dependent kinases (CDKs) that are activated by cyclins. In order to determine the influence of the tested compounds on the expression of different factors implicated in the regulation of the cell cycle, the expression of *cyclin A*, *cyclin B*, *CDK1* (Cyclin-dependent kinase 1), and *CDC20* (cell division cycle protein 20) genes was analyzed (Figure 7). Compounds **4** and **5** slightly reduced *cyclin A*, *CDK1*, and *CDC20* gene expression and induced the expression of the *cyclin B* gene. Compounds **2** and **10** reduced *cyclin A*, *cyclin B*, and *CDC20* gene expression and induced the expression of the *CDK1* gene. Compound **6** proved to have a weak influence on the expression of genes implicated in the cell cycle by slightly increasing the expression of *cyclin A*, *cyclin B*, and *CDK1* genes and slightly reducing the expression of *CDC20*. Compounds **1**, **7**, **8**, **9**, and **11** reduced the expression levels of *cyclin A*, *cyclin B*, *CDK1*, and *CDC20* genes, indicating an inhibition of cell cycle progression, which is in agreement with the decrease of S and G2 phases demonstrated by flow cytometry. Compounds **3**, **12**, and **13** increased the evaluated genes expressions. The influence of these compounds on the gene expression of *cyclin B* and *CDK1* could explain no apoptotic effect observed disregard increased levels of expression of caspases. CDK1/cyclin B complex could phosphorylate procaspase 8, inhibiting its processing and, thus, the activation of apoptosis through extrinsic pathway [62], and, also, could phosphorylate caspase 9 at an inhibitory site [63], consequently inhibiting the intrinsic apoptotic pathway. On the other hand, CDK1/cyclin B could also target p53, its Ser-315 phosphorylation enhancing ATP generation and membrane potential in mitochondria [64].

The blocking in the G1 phase observed after 24 h of treatment with some isoniazid derivatives was previously reported in the literature [65]. The influence of these compounds on the cell cycle was explained by their activity as Fe-chelators, determining cyclin D proteolysis and cyclin E accumulation, and a decrease of CDK2 (Cyclin-dependent kinase 2) level [65,66].

In the current study, compounds **7**–**9** reduced the expression levels of genes implicated in S, G2, and M phases of cell cycle and caused cell cycle blocking in G0/G1 phase. This effect can also be explained by their metabolism (discussed above), with presumed metabolites being capable of forming complexes that could inactivate enzymes involved in abnormal cell division [57].

The influence of compounds **7**–**9** on cell cycle indicates their antitumor potential and further studies on their mechanism of action should/will be done.

## 3. Materials and Methods

### 3.1. Chemicals and Analytical Techniques

All chemicals and reagents were used as purchased from commercial suppliers (MilliporeSigma, Burlington, MA, USA and Chimopar, Bucharest, Romania, respectively) and were of the highest available purity. The solvents were used as absolute solvents, as well.

Analytical thin-layer chromatography (TLC) was performed on aluminium TLC plates that were silica gel-coated with fluorescent indicator F254, with size 20 × 20 cm, (Merck KGaA, Darmstadt, Germany). The spots were visualized using a fixed wavelength (254 nm) UV light. 

The ^1^H(^13^C)-NMR spectra, in deuterated solvents DMSO-d_6_/CDCl_3_ (isotopic purity 99.9%), were recorded on Varian Inova-400 NMR spectrometer (Agilent, Santa Clara, CA, USA, former Varian Inc., Palo Alto, CA, USA) of 400 MHz (^1^H)and 100 MHz (^13^C), equipped with a Varian broadband direct (^1^H/X) probe. The standard for chemical shift is tetramethylsilane (TMS) and J-values were calculated in Hz. Chemical shifts δ [ppm] are referenced to protonated solvent signals as internal standard DMSO-d_6_: δ = 2.50 ppm (^1^H), 39.52 ppm (^13^C), and CDCl_3_: δ = 7.26 ppm (^1^H), 77.16 ppm (^13^C) [63,64,65,66]. Signal multiplicities are abbreviated as *s* (singlet), *d* (doublet), *dd* (doublet of doublet), *t* (triplet), *dt* (doublet of triplet), *q* (quadruplet), and *m* (multiplet) with the prefix *b* in case of broad signals [67,68,69,70].

Molecular identification of organic functional groups in solid sample was performed by Fourier transform infrared spectroscopy using a Vertex 80v spectrometer (Bruker, Ettlingen, Germany), equipped with attenuated total reflectance (ATR) accessory. FTIR spectra were recorded in the range of 4000 to 400 cm^−1^, with 0.2 cm^−1^ spectral resolution, 0.1% T accuracy, and 32 scans/spectra.

Melting points were determined on a melting point meter Krüss M5000 (A. Krüss Optronic, Hamburg, Germany), up to 400 °C, and are given uncorrected.

Mass spectra were recorded on a Maxis Bruker 4G spectrometer (Bruker Corporation, Billerica, MA, USA) with an electrospray ionization source (ESI). For the biological approach, the synthetized compounds were initially dissolved in DMSO (i.e., 1 mg/mL) to prepare a concentrated stock solution. Finally, additional dilutions (e.g., 1 μg/mL in methanol) were prepared in culture medium. The scanning range of molecular ions (*m*/*z*) was 50 to 1250. 

### 3.2. Synthesis and Characterization of Isoniazid Derivatives 

#### 3.2.1. *N*,*N*’-diacylhydrazines and *N*,*N*,*N*’-triacylhydrazines 

**Figure 8:**
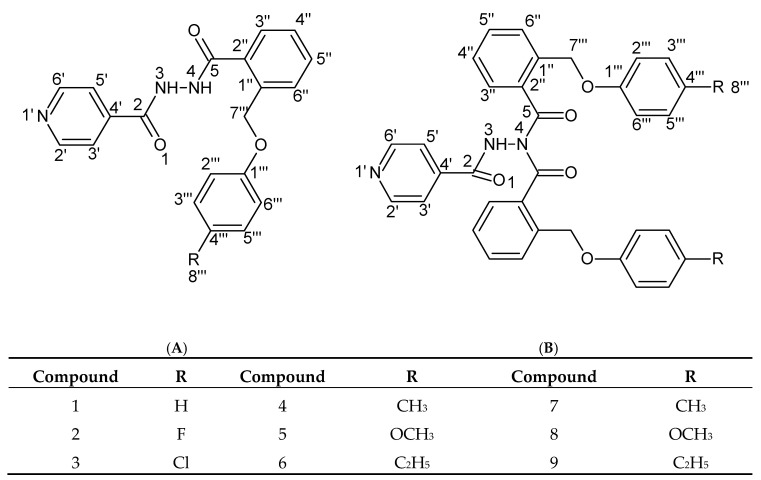
General formula of newly synthesized *N,N’*-diacylhydrazines (compounds **1**–**6**) (**A**) and *N,N,N’*-triacylhydrazines (compounds **7**–**9**) (**B**).

To 5 mmol of 2-(4-substituted-phenoxymethyl)-benzoic acid dissolved in 25 mL of 1,2-dichloroethan was added 10 mmol of thionyl chloride. The mixture was refluxed for 3 h. The excess of thionyl chloride and 1,2-dichloroethan was removed under reduced pressure. A quantity of 5 mmol of isoniazid dissolved in 30 mL of dichloromethane was added to the crude acid chloride and the mixture was stirred overnight at room temperature. The next day, a double volume of water was added to the reaction mixture and the solid formed was separated by filtration and washed twice with dichloromethane. After filtration, the aqueous phase was extracted three times with dichloromethane and the organic layer was dried over anhydrous sodium sulfate. The solvent was removed under reduced pressure and the products (Figure 8) were separated and purified on chromatographic columns filled with silica gel using dichloromethane: ethyl acetate in gradient for elution. 

##### Compound **1**

*N*-(2-Phenoxymethyl-benzoyl)-*N*’-(isonicotinoyl)-hydrazine 

Yield: 52%

M.p.: 128–129 °C

Elemental analysis: C_20_H_17_N_3_O_3_, M = 347; calcd: C = 69.16%, H = 4.9%, N = 12.1%; found: C = 69.14%, H = 4.93%, N = 12.08%.

^1^H-NMR (400 MHz, DMSO-d_6_, δ ppm, *J* Hz): 10.93 (s, 1H, H-3), 10.52 (s, 1H, H-4), 8.89 (d, 2H, H-2′, H-6′, 5.9), 7.96 (d, 1H, H-3′′, 7.4), 7.81(d, 2H, H-3′, H-5′, 6.0), 7.63 (t, 1H, H-5′′, 7.3), 7.54 (d, 1H, H-6′′,7.8), 7.30 (t, 1H, H-4′′, 7.4), 7.34 (t, 2H, H-3′′′, H-5′′′, 8.0), 7.03-6.99 (m, 3H, H-2′′′, H-4′′′, H-6′′′), 5.21 (s, 2H, H-7′′′).

^13^C-NMR (100 MHz, DMSO-d_6_, δ ppm): 167.3 (C-5), 164.5 (C-2), 159.0 (C-1′′′), 149.7 (C-2′, C-6′), 140.8 (C-4′), 136.5 (C-1′′), 133.6 (C-2′′), 132.3 (C-5′′), 129.7 (C-3′′′, C-5′′′), 128.5 (C-4′′, 127.7 (C-3′′), 124.6 (C-6′′), 121.7 (C-3′, C-5′), 121.0 (C-4′′′), 114.3 (C-2′′′, C-6′′′), 68.5 (C-7′′′).

FT-IR (ATR in solid, *ν* cm^−1^): 3199; 3030; 2915; 2468; 1672; 1607; 1588; 1547; 1510; 1455; 1282; 1240; 1140; 815; 742.

HRMS *m*/*z* 348.1346 [M + H]^+^ (calcd for C_20_H_18_N_3_O_3_^+^: 348.1342).

*R_f_* (silicagel, AcOEt:MeOH = 9:1): 0.50.

##### Compound **2**

*N*-[2-(p-Fluoro-phenoxymethyl)-benzoyl]-*N*’-(isonicotinoyl)-hydrazine 

Yield: 40%

M.p.: 168–169 °C

Elemental analysis: C_20_H_16_FN_3_O_3_, M = 365; calcd: C = 65.75%, H = 4.38%, N = 11.51%; found: C = 65.73%, H = 4.39%, N = 11.53%.

^1^H-NMR (400 MHz, DMSO-d_6_, δ ppm, *J* Hz): 10.98 (s, 1H, H-3), 10.56 (s, 1H, H-4), 8.87 (d, 2H, H-2′, H-6′, 6.0), 7.95 (d, 2H, H-3′, H-5′, 5.92), 7.66 (m, 2H, H-3′′, H-6′′), 7.57 (t, 1H, H-5′′, 7.18), 7.51 (t, 1H, H-4′′, 7.41), 7.04 (d, 2H, H-2′′′, H-6′′′, 8.4), 6.9 (d, 2H, H-3′′′, H-5′′′, 8.4), 5.35 (s, 2H, H-7′′′).

^13^C-NMR (100 MHz, DMSO-d_6_, δ ppm): 167.9 (C-5), 164.2 (C-2), 162.1 (C-4′′′), 156.4 (C-1′′′), 150.0 (C-2′, C-6′), 140.8 (C-4′), 136.5 (C-1′′), 133.4 (C-2′′), 131.1 (C-5′′), 129.5 (C-4′′), 127.5 (C-3′′), 127.3 (C-6′′), 122.2 (C-3′, C-5′), 116.2 (C-3′′′, C-5′′′), 114.5 (C-2′′′, C-6′′′), 66.5 (C-7′′′).

FT-IR (ATR in solid, *ν* cm^−1^): 3242; 3037; 2923; 2451; 1689; 1598; 1581; 1524; 1443; 1237; 1134; 1114; 828; 739.

HRMS *m*/*z* 366.1248 [M + H]^+^ (calcd for C_20_H_17_FN_3_O_3_^+^: 366.1248).

*R_f_* (silicagel, AcOEt:MeOH = 9:1): 0.57.

##### Compound **3**

*N*-[2-(p-Chloro-phenoxymethyl)-benzoyl]-*N’*-(isonicotinoyl)-hydrazine 

Yield: 25%

M.p.: 145–146 °C

Elemental analysis: C_20_H_16_ClN_3_O_3_, M = 381.5; calcd: C = 62.91%, H = 4.19%, N = 11.01%; found: C = 62.89%, H = 4.16%, N = 11.03%.

^1^H-NMR (400 MHz, DMSO-d_6_, δ ppm, *J* Hz): 11.2 (s, 1H, H-3), 10.96 (s, 1H, H-4), 9.12 (d, 2H, H-2′, H-6′, 5.95), 8.34 (d, 2H, H-3′, H-5′, 5.8), 7.99 (m, 2H, H-3′′, H-6′′), 7.81 (t, 1H, H-5′′, 7.0), 7.76 (t, 1H, H-4′′, 7.32), 7.5 (d, 2H, H-3′′′, H-5′′′, 8.0), 6.70 (d, 2H, H-2′′′, H-6′′′, 8.1), 5.74 (s, 2H, H-7′′′).

^13^C-NMR (100 MHz, DMSO-d_6_, δ ppm): 167.9 (C-5), 164.4 (C-2), 156.6 (C-1′′′), 150.1 (C-2′, C-6′), 140.7 (C-4′), 136.6 (C-1′′), 133.7 (C-2′′), 131.4 (C-5′′), 125.1 (C-4′′′), 129.9 (C-4′′), 129.2 (C-3′′′, C-5′′′), 128.2 (C-3′′), 127.8 (C-6′′), 122.2 (C-3′, C-5′), 115.4 (C-2′′′, C-6′′′), 67.1 (C-7′′′).

FT-IR (ATR in solid, *ν* cm^−1^): 3215; 3037; 2968; 1690; 1598; 1573; 1510; 1452; 1247; 1218; 1139; 826; 741; 713.

HRMS *m*/*z* 382.0954 [M + H]^+^ (calcd for C_20_H_17_ClN_3_O_3_^+^: 382.0952).

*R_f_* (silicagel, AcOEt:MeOH = 9:1): 0.56.

##### Compound **4**

*N*-[2-(p-Tolyloxymethyl)-benzoyl]-*N*’-(isonicotinoyl)-hydrazine 

Yield: 58%

M.p.: 173–175 °C

Elemental analysis: C_21_H_19_N_3_O_3_, M = 361; calcd: C = 69.81%, H = 5.26%, N = 11.63%; found: C = 69.83%, H = 5.25%, N = 11.65%.

^1^H-NMR (400 MHz, DMSO-d_6_, δ ppm, *J* Hz): 10.96 (s, 1H, H-3), 10.55 (s, 1H, H-4), 8.84 (d, 2H, H-2′, H-6′, 6.05), 7.91 (d, 2H, H-3′, H-5′, 5.86), 7.62 (m, 2H, H-3′′, H-6′′), 7.55 (t, 1H, H-5′′, 7.22), 7.46 (t, 1H, H-4′′, 7.42), 7.08 (d, 2H, H-3′′′, H-5′′′, 8.2), 6.90 (d, 2H, H-2′′′, H-6′′′, 8.39), 5.30 (s, 2H, H-7′′′), 2.22 (s, 3H, H-8′′′).

^13^C-NMR (100 MHz, DMSO-d_6_, δ ppm): 167.5 (C-5), 163.9 (C-2), 156.1 (C-1′′′), 149.6 (C-2′, C-6′), 140.2 (C-4′), 136.0 (C-1′′), 133.0 (C-2′′), 130.5 (C-5′′), 129.8 (C-4′′′), 129.5 (C-4′′), 127.8 (C-3′′′, C-5′′′), 127.6 (C-3′′), 127.5 (C-6′′), 121.8 (C-3′, C-5′), 114.6 (C-2′′′, C-6′′′), 66.5 (C-7′′′), 20.0 (C-8′′′).

FT-IR (ATR in solid, *ν* cm^−1^): 3191; 3027; 2919; 2461; 1669; 1607; 1590; 1551; 1510; 1455; 1291; 1244; 1140; 811; 739. 

MS *m*/*z* 362.1023 [M + H]^+^ (calcd for C_21_H_20_N_3_O_3_^+^: 362.4018).

*R_f_* (silicagel, AcOEt:CH_2_Cl_2_ = 1:1):0.16.

##### Compound **5**

*N*-[2-(p-Methoxy-phenoxymethyl)-benzoyl]-*N*’-(isonicotinoyl)-hydrazine 

Yield: 54%

M.p.: 108–111 °C

Elemental analysis: C_21_H_19_N_3_O_4_, M = 377; calcd: C = 66.84%, H = 5.04%, N = 11.17%; found: C = 66.82%, H = 5.02%, N = 11.17%.

^1^H-NMR (400 MHz, CDCl_3_, δ ppm, *J* Hz): 10.11 (s, 1H, H-3), 9.72 (s, 1H, H-4), 8.63 (d, 2H, H-2′, H-6′, 5.5), 7.74 (d, 1H, H-3′′, 7.62), 7.62 (d, 2H, H-3′, H-5′, 5.67), 7.52 (m, 2H, H-5′′, H-6′′), 7.42 (t, 1H, H-4′′, 6.64), 6.93 (d, 2H, H-3′′′, H-5′′′, 8.98), 6.78 (d, 2H, H-2′′′, H-6′′′, 8.98), 5.22 (s, 2H, H-7′′′), 3.73 (s, 3H, H-8′′′).

^13^C-NMR (100 MHz, CDCl_3_, δ ppm): 166.5 (C-5), 162.8 (C-2), 154.7 (C-1′′′), 152.2 (C-4′′′), 150.6 (C-2′, C-6′), 138.5 (C-4′), 135.7 (C-1′′), 132.1 (C-2′′), 131.8 (C-3′′), 130.4 (C-5′′), 129.0 (C-4′′), 128.7 (C-6′′), 121.0 (C-3′, C-5′), 116.61 (C-3′′′, C-5′′′), 114.82 (C-2′′′, C-5′′′), 69.55 (C-7′′′), 55.72 (C-8′′′).

FT-IR (ATR in solid, *ν* cm^−1^): 3199; 3066; 2993; 1683; 1592; 1576; 1550; 1452; 1242; 1213; 1140; 1038; 830; 743.

MS *m*/*z* 378.0935 [M + H]^+^ (calcd for C_21_H_20_N_3_O_4_^+^: 378.4012).

*R_f_* (silicagel, AcOEt:CH_2_Cl_2_ = 1:1): 0.13.

##### Compound **6**

*N*-[2-(p-Ethyl-phenoxymethyl)-benzoyl]-*N*’-(isonicotinoyl)-hydrazine 

Yield: 33%

M.p.: 133–135 °C

Elemental analysis: C_22_H_21_N_3_O_3_, M = 375; calcd: C = 70.40%, H = 5.60%, N = 11.20%; found: C = 70.36%, H = 5.62%, N = 11.23%.

^1^H-NMR (400 MHz, DMSO-d_6_, δ ppm, *J* Hz): 10.90 (s, 1H, H-3), 10.53 (s, 1H, H-4), 8.78 (d, 2H, H-2′, H-6′, 3.91), 7.83 (d, 2H, H-3′, H-5′, 4.30), 7.60 (m, 2H, H-3′′, H-6′′), 7.53 (t, 1H, H-5′′,7.23), 7.43 (t, 1H, H-4′′, 7.22), 7.09 (d, 2H, H-3′′′, H-5′′′, 8.01), 6.90 (d, 2H, H-2′′′, H-6′′′, 8.01), 5.29 (s, 2H, H-7′′′), 2.50 (m, 2H, overlapping with DMSO-d6 signal, H-8′′′), 1.11 (t, 3H, H-9′′′, 7.42).

^13^C-NMR (100 MHz, DMSO-d_6_, δ ppm): 167.6 (C-5), 164.2 (C-2), 156.3 (C-1′′′), 150.5 (C-2′, C-6′), 139.4 (C-4′), 136.1 (C-1′′), 136.0 (C-4′′′), 133.1 (C-2′′), 130.6 (C-5′′), 128.7 (C-4′′), 127.9 (C-3′′′, C-5′′′), 127.6 (C-3′′), 127.6 (C-6′′), 121.4 (C-3′, C-5′), 114.7 (C-2′′′, C-2′′′), 66.5 (C-7′′′), 27.3 (C-8′′′), 15.9 (C-9′′′).

FT-IR (ATR in solid, *ν* cm^−1^): 3265; 3030; 2961; 2926; 2870; 1690; 1608; 1581; 1509; 1453; 1218; 1140; 827; 736.

MS *m*/*z* 376.0836 [M + H]^+^ (calcd for C_22_H_22_N_3_O_3_^+^: 376.3283).

*R_f_* (silicagel, AcOEt:CH_2_Cl_2_ = 1:1): 0.17.

##### Compound **7**

*N*,*N*-di[2-(p-Tolyloxymethyl)-benzoyl]-*N*’-(isonicotinoyl)-hydrazine

Yield: 5%

M.p.: semisolid

Elemental analysis: C_36_H_31_N_3_O_5_, M = 585; calcd: C = 73.85%, H = 5.30%, N = 7.18%; found: C = 73.82%, H = 5.28%, N = 7.19%.

^1^H-NMR (400 MHz, DMSO-d_6_, δ ppm, *J* Hz): 11.85 (s, 1H, H-3), 8.77 (d, 2H, H-2′, H-6′, 5.86), 7.71 (d, 2H, H-3′′, 7.42), 7.57 (m, 4H, H-3′, H-5′′, H-6′′), 7.50 (t, 2H, H-5′′, 7.20), 7.38 (t, 2H, H-4′′, 6.83), 7.04 (d, 4H, H-5′′′, H-3′′′, 8.59), 6.87 (d, 4H, H-2′′′, H-6′′′, 8.40), 5.20 (s, 4H, H-7′′′), 2.21 (s, 6H, H-8′′′).

^13^C-NMR (400 MHz, DMSO-d_6_, δ ppm): 169.8(C-5), 164.6 (C-2), 155.9 (C-1′′′), 150.5 (C-2′, C-6′), 138.2 (C-4′), 136.1 (C-1′′), 132.6 (C-2′′), 131.3 (C-5′′), 129.7 (C-4′′′), 129.5 (C-4′′), 127.9 (C-3′, C-5′), 127.4 (C-3′′), 127.2 (C-6′′), 121.0 (C-3′′′, C-5′′′), 114.6 (C-2′′′, C-6′′′), 66.4 (C-7′′′), 20.0 (C-8′′′).

FT-IR (ATR in solid, *ν* cm^−1^): 3271; 3030; 2923; 1689; 1609; 1583; 1508; 1217; 1140; 811; 739.

MS *m*/*z* 586.2167 [M + H]^+^ (calcd for C_36_H_32_N_3_O_5_^+^: 586.5563).

*R_f_* (silicagel, AcOEt:CH_2_Cl_2_ = 1:1): 0.60.

##### Compound **8**

*N*,*N*-di[2-(p-Methoxy-phenoxymethyl)-benzoyl]-*N*’-(isonicotinoyl)-hydrazine

Yield: 2%

M.p.: semisolid

Elemental analysis: C_36_H_31_N_3_O_7_, M = 617; calcd: C = 70.02%, H = 5.02%, N = 6.81%; found: C = 70.05%, H = 5.00%, N = 6.79%.

^1^H-NMR (400 MHz, CDCl_3_, δ ppm, *J* Hz): 8.62 (d, 2H, H-2′, H-6′, 5.47), 8.53 (s, 1H, H-3), 7.69 (d, 1H, H-3′′, 7.81), 7.55 (d, 2H, H-6′′, 7.62), 7.45 (t, 2H, H-5′′, 7.48), 7.33 (t, 2H, H-4′′, 7.61), 7.15 (d, 2H, H-3′, H-5′, 5.86), 6.88 (d, 4H, H-3′′′, H-5′′′, 9.18), 6.78 (d, 4H, H-2′′′, H-6′′′, 8.98), 5.19 (s, 4H, H-7′′′), 3.72 (s, 6H, H-8′′′).

^13^C-NMR (100 MHz, CDCl_3_, δ ppm): 170.4 (C-5), 164.4 (C-2), 154.4 (C-1′′′), 152.4 (C-4′′′), 150.5 (C-2′, C-6′), 138.7(C-4′), 135.5 (C-1′′), 133.5 (C-2′′), 131.4 (C-5′′), 131.1 (C-4′′), 128.7 (C-3′′), 126.7 (C-6′′), 120.8 (C-3′, C-5′), 116.8 (C-3′′′, C-5′′′), 114.9 (C-2′′′, C-6′′′), 68.6 (C-7′′′), 55.7 (C-8′′′).

FT-IR (ATR in solid, *ν* cm^−1^): 3267; 3065; 2932; 1688; 1556; 1503; 1463; 1207; 1139; 1032; 821; 737.

MS *m*/*z* 618.2467 [M + H]^+^ (calcd for C_36_H_32_N_3_O_7_^+^: 618.5551).

*R_f_* (silicagel, AcOEt:CH_2_Cl_2_ = 1:1): 0.52.

##### Compound **9**

*N,N*-di[2-(p-Ethyl-phenoxymethyl)-benzoyl]-*N*’-(isonicotinoyl)-hydrazine

Yield: 6%

M.p.: semisolid

Elemental analysis: C_38_H_35_N_3_O_5_, M = 613; calcd: C = 74.39%, H = 5.71%, N = 6.85%; found: C = 74.37%, H = 5.69%, N = 6.87%.

^1^H-NMR (DMSO, δ ppm, *J* Hz): 11.97 (s, 1H, H-3), 8.77 (d, 2H, H-2′, H-6′, 4.69), 7.73 (d, 2H, H-3′′, 7.62), 7.58 (m, 4H, H-3′, H-5′, H-6′′), 7.50 (t, 2H, H-5′′, 7.42), 7.37 (t, 2H, H-4′′, 7.03), 7.07 (d, 4H, H-3′′′, H-5′′′, 8.01), 6.90 (d, 4H, H-2′′′, H-6′′′, 8.20), 5.21 (s, 4H, H-7′′′), 2.50 (m, 4H, overlapping with DMSO-d6 signal, H-8′′′), 1.12 (t, 6H, H-9′′′, 7.43).

^13^C-NMR (DMSO, δ ppm): 169.9 (C-5), 164.7 (C-2), 156.1 (C-1′′′), 150.5 (C-2′, C-6′), 138.5 (C-4′), 136.1 (C-1′′), 132.7 (C-4′′′), 131.2 (C-2′′), 129.6 (C-5′′), 128.6 (C-4′′), 128.0 (C-3′′′, C-5′′′), 127.4 (C-3′′), 127.2 (C-6′′), 121.1 (C-3′, C-5′), 114.6 (C-2′′′, C-6′′′), 66.4 (C-7′′′), 27.3 (C-8′′′), 15.8 (C-9′′′).

FT-IR (ATR in solid, *ν* cm^−1^): 3231; 3022; 2958; 2923; 1674; 1628; 1599; 1554; 1507; 1476; 1244; 1134; 831; 748.

MS *m*/*z* 614.0954 [M + H]^+^ (calcd for C_38_H_36_N_3_O_5_^+^: 614.3095).

*R_f_* (silicagel, AcOEt:CH_2_Cl_2_ = 1:1): 0.63.

#### 3.2.2. 1,3,4-Oxadiazoles Synthesis

**Figure 9:**
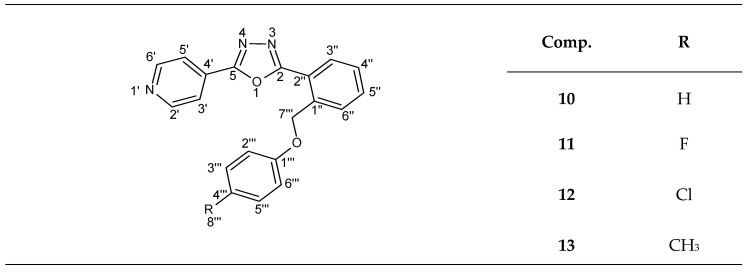
General formula of newly synthesized 1,3,4-oxadiazoles (compounds **10**–**13**).

A quantity of 1 mmol *N*,*N*’-diacylhydrazine was suspended in 15 mL toluene. After 20 min of stirring, 5 mmol phosphoryl chloride was added dropwise. The reaction mixture was refluxed for 6 h and stirred overnight at room temperature. The solvent and residual phosphoryl chloride were removed under reduced pressure. The residue was taken up in dichloromethane and washed three times with 5% NaOH solution and three times with saturated aqueous NaCl solution. The organic layer was dried over anhydrous sodium sulfate and distilled under reduced pressure to remove the solvent. The final compound (Figure 9) was recrystallized using ethanol.

##### Compound **10**

22-[2-(Phenoxymethyl)-phenyl]-5-(pyridine-4-yl)-1,3,4-oxadiazole

Yield: 62%

M.p.: 210–211 °C

Elemental analysis: C_20_H_15_N_3_O_2_, M = 329; calcd: C = 72.95%, H = 4.56%, N = 12.77%; found: C = 72.97%, H = 4.58%, N = 12.74%.

^1^H-NMR (400 MHz, DMSO-d6, δ ppm, *J* Hz): 8.87 (d, 2H, H-2′, H-6′, 4.00), 8.24 (d, 1H, H-3′′, 8.00), 8.05 (d, 2H, H-3′, H-5′, 4.00), 7.80 (d, 1H, H-6′′, 8.00), 7.72 (t, 1H, H-5′′, 7.99), 7.62 (t, 1H, H-4′′, 7.99), 7.29 (d, 2H, H-3′′′, H-5′′′, 8.00), 7.02-6.93 (m, 3H, H-2′′′, H-4′′′, H-6′′′), 5.58 (s, 2H, H-7′′′).

^13^C-NMR (100 MHz, DMSO-d6, δ ppm): 164.8 (C-5), 162.1(C-2), 158.2(C-1′′′), 149.2 (C-2′, C-6′), 136.7 (C-4′), 132.3 (C-5′′), 132.0 (C-2′′), 130.4 (C-4′′), 129.6 (C-3′′′, C-5′′′), 129.2 (C-6′′), 128.6 (C-3′′), 121.4 (C-1′′),121.1 (C-4′′′), 120.0 (C-3′, C5′), 114.5 (C-2′′′, C-6′′′), 67.6 (C-7′′′).

FT-IR (ATR in solid, *ν* cm^−1^): 3030; 2964; 2815; 2160; 1604; 1574; 1552; 1503; 1452; 1232; 1125; 1015; 824; 742.

HRMS *m*/*z* 330.1237 [M + H]^+^ (calcd for C_20_H_16_N_3_O_2_^+^: 330.1237).

*R_f_* (silicagel, AcOEt:MeOH = 9:1): 0.72.

##### Compound **11**

22-[2-(p-Fluoro-phenoxymethyl)-phenyl]-5-(pyridine-4-yl)-1,3,4-oxadiazole

Yield: 65%

M.p.: 298–299 °C

Elemental analysis: C_20_H_14_FN_3_O_2_, M = 347; calcd: C = 69.16%, H = 4.03%, N = 12.1%; found: C = 69.15%, H = 4.05%, N = 12.12%.

^1^H-NMR (400 MHz, DMSO-d6, δ ppm*, J* Hz): 8.74 (d, 2H, H-2′, H-6′, 4.68), 8.21 (d, 1H, H-3′′, 7.3), 7.84 (d, 2H, H-3′, H-5′, 5.7), 7.75 (d, 1H, H-6′′, 7.42), 7.67 (t, 1H, H-5′′, 6.6), 7.57 (t, 1H, H-4′′, 7.6), 6.87 (d, 2H, H-3′′′, H-5′′′, 8.21), 7.03 (d, 2H, H-2′′′, H-6′′′, 8.2), 5.51 (s, 2H, H-7′′′).

^13^C-NMR (100 MHz, DMSO-d6, δ ppm): 164.9 (C-5), 162.8 (C-2), 156.6 (C-1′′′), 152.1 (C-4′′′), 151.3 (C-2′, C-6′), 137.2 (C-4′), 132.5 (C-5′′), 131.0 (C-2′′), 129.9 (C-1′′), 129.8 (C-3′′), 129.5 (C-6′′), 129.0 (C-4′′), 120.7 (C-3′, C5′), 115.2 (C-3′′′, C-5′′′), 114.9 (C-2′′′, C-6′′′), 68.3 (C-7′′′).

FT-IR (ATR in solid, *ν* cm^−1^): 3029; 2974; 2844; 2163; 1608; 1581; 1508; 1455; 1241; 1124; 1114; 1015; 841; 739.

HRMS *m*/*z* 348.1145 [M + H]^+^ (calcd for C_20_H_15_FN_3_O_2_^+^: 348.1142).

*R_f_* (silicagel, AcOEt:MeOH = 9:1): 0.79.

##### Compound **12**

22-[2-(p-Chloro-phenoxymethyl)-phenyl]-5-(pyridine-4-yl)-1,3,4-oxadiazole

Yield: 68%

M.p.: 341–342 °C

Elemental analysis: C_20_H_14_ClN_3_O_2_, M = 363.5; calcd: C = 66.02%, H = 3.85%, N = 11.55%; found: C = 66.01%, H = 3.87%, N = 11.57%.

^1^H-NMR (400 MHz, DMSO-d6, δ ppm, *J* Hz): 8.71 (d, 2H, H-2′, H-6′, 4.68), 8.14 (d, 1H, H-3′′, 7.3), 7.81 (d, 2H, H-3′, H-5′, 5.82), 7.73 (d, 1H, H-6′′, 7.35), 7.64 (t, 1H, H-5′′, 6.65), 7.58 (t, 1H, H-4′′, 7.69), 7.52 (d, 2H, H-3′′′, H-5′′′, 8.39), 6.74 (d, 2H, H-2′′′, H-6′′′, 8.39), 5.49 (s, 2H, H-7′′′).

^13^C-NMR (100 MHz, DMSO-d6, δ ppm): 164.1 (C-5), 161.9 (C-2), 155.9 (C-1′′′), 150.3 (C-2′, C-6′), 136.5 (C-4′), 132.0 (C-5′′), 130.4 (C-2′′), 129.4 (C-1′′), 129.2 (C-3′′), 129.0 (C-6′′), 128.7 (C-3′′′, C-5′′′), 128.1 (C-4′′), 124.8(C-4′′′), 120.0 (C-3′, C5′), 114.8 (C-2′′′, C-6′′′), 67.7 (C-7′′′).

FT-IR (ATR in solid, *ν* cm^−1^): 3030; 2991; 2843; 2159; 1604; 1582; 1551; 1510; 1455; 1218; 1125; 840; 745; 713.

HRMS *m*/*z* 364.0837 [M + H]^+^ (calcd for C_20_H_15_ClN_3_O_2_^+^: 364.2827).

*R_f_* (silicagel, AcOEt:MeOH = 9:1): 0.75.

##### Compound **13**

22-[2-(p-Tolyloxymethyl)-phenyl]-5-(pyridine-4-yl)-1,3,4-oxadiazole

Yield: 73%

M.p.: 249–250 °C

Elemental analysis: C_21_H_17_N_3_O_2_, M = 343; calcd: C = 73.47%, H = 4.96%, N = 12.24%; found: C = 73.45%, H = 4.93%, N = 12.27%.

^1^H-NMR (400 MHz, DMSO-d6, δ ppm, *J* Hz): 8.79 (d, 2H, H-2′, H-6′, 4.71), 8.22 (d, 1H, H-3′′, 7.29), 7.89 (d, 2H, H-3′, H-5′, 5.73), 7.78 (d, 1H, H-6′′, 7.40), 7.70 (t, 1H, H-5′′, 6.63), 7.62 (t, 1H, H-4′′, 7.69), 7.08 (d, 2H, H-3′′′, H-5′′′, 8.35), 6.87 (d, 2H, H-2′′′, H-6′′′, 8.44), 5.53 (s, 2H, H-7′′′), 2.21 (s, 3H, H-8′′′).

^13^C-NMR (100 MHz, DMSO-d6, δ ppm): 164.5 (C-5), 162.4 (C-2), 156.1 (C-1′′′), 150.8 (C-2′, C-6′), 136.8 (C-4′), 132.2 (C-5′′), 130.4 (C-2′′), 129.9 (C-3′′′, C-5′′′), 129.6 (C-1′′), 129.5 (C-3′′), 129.1 (C-6′′), 128.5 (C-4′′), 121.4 (C-4′′′), 120.2 (C-3′, C5′), 114.5 (C-2′′′, C-6′′′), 67.7 (C-7′′′), 20.1 (C-8′′′).

FT-IR (ATR in solid, *ν* cm^−1^): 3028; 2924; 2870; 2158; 1608; 1587; 1550; 1509; 1457; 1240; 1125; 810; 735.

HRMS *m*/*z* 344.1394 [M + H]^+^ (calcd for C_21_H_18_N_3_O_2_^+^: 344.3465).

*R_f_* (silicagel, CH_2_Cl_2_:MeOH = 5:1): 0.60.

### 3.3. Anti-Mycobacterial Activity

The *M. tuberculosis* clinical strain used for anti-mycobacterial assay was maintained on Middlebrook 7H9 broth. The anti-mycobacterial activity was evaluated by the microplate Alamar Blue assay (MABA) as stated by Franzblau et al. [71]. We used serial dilutions of the new synthesized compounds and isoniazid, the final drug concentrations being between 25 μg/mL to 0.012 μg/mL. Equal volumes of *M. tuberculosis* inoculum and tested compounds were mixed and incubated at 37 °C for eight days in 96-well plates covered and sealed with parafilm. On day nine, a volume of 10 µL of freshly prepared mixture (1:1) of Alamar Blue reagent (0.1 mg/mL) and tween 80 (10%) was added to each well and kept at 37 °C for another 24 h. A blue color in the well was interpreted as no bacterial growth, and a pink color was scored as growth. The MIC (Minimal Inhibition Concentration) was defined as the lowest drug concentration, which prevented a color change from blue to pink.

### 3.4. Antibacterial Activity

The antimicrobial activity of the newly synthesized compounds was determined according to Matei et al. [51], using the following strains: *Acinetobacter baumanii*, *Citrobacter koseri*, *Escherichia coli*, *Enterobacter cloacae*, *Enterococcus faecalis*, *Klebsiella pneumonia*, *Morganella morganii*, *Pseudomonas aeruginosa*, *Peudomonas putida*, *Salmonella typhimurium*, *Serratia marcescens*, *Shigella sonnei*, *Stenotrophomonas maltophilia*, *Staphylococcus hominis*, *Staphylococcus aureus*, and coagulase-negative staphylococci, using an adapted agar disk diffusion technique. Briefly, bacterial suspensions of 0.5 McFarland density obtained from 24 h cultures were seed on solid medium. Subsequent, a volume of 5 μL of tested compounds (1 mg/mL solutions) were spotted on inoculated plates. Anti-bacterial activity was assessed after incubation for 24 h at 37 °C.

### 3.5. Cell Culture

Two cell lines, HCT-8 (CCL-224) and HT-29 (ATCC-HTB-38), were used to evaluate the activity of new compounds on eukaryotic cells. These cells were maintained as adherent cultures in Dulbecco′s Modified Essential Medium (DMEM) (Sigma, Ronkonkoma, NY, USA) supplemented with 10% heat-inactivated fetal bovine serum (Sigma, USA) at 37 °C, 5% CO_2_, in a humid atmosphere.

### 3.6. Apoptosis Detection

The apoptotic effect of tested compounds was evaluated using Annexin V-FITC Apoptosis Detection Kit I (BD Bioscience Pharmingen, USA) according to manufacturer protocol. In this regard, HT-29 cells (3 × 10^6^), were seeded in 3.5 cm diameter wells and treated with 50 µg/mL of tested compounds. After 24 h, the cells were resuspended in 100 µL of binding buffer and stained with 5 µL Annexin V-FITC and 5 µL propidium iodide for 10 min in dark. At least 10,000 events from each sample were acquired using a Beckman Coulter flow cytometer. The analysis was done using FlowJo v 8.8.6 software (Ashland, OR, USA).

### 3.7. Cell Cycle Analysis

To assess the influence of new compounds on cell cycle, a number of 3 × 10^6^ HT-29 cells were treated with compounds at 50 µg/mL concentration for 24 h. Treated cells were harvested, washed in a cold phosphate-buffered saline solution (PBS), then fixed in cold ethanol (70%) and stored at − 20 °C. After 24 h, the cells were washed with PBS, resuspended in 100 µl PBS, treated with 1 mg/mL RNase A and labeled with 100 μg/mL propidium iodide in the dark at room temperature for 30 min. DNA content of cells was quantified on a Beckman Coulter EPICS XL flow cytometer and analyzed using FlowJo 8.8.6 software (Ashland, OR, USA).

### 3.8. Quantitative RT-PCR for Analysis of Genes Expression

A number of 3 × 10^6^ HT-29 or HCT-8 cells treated with isoniazid and compounds **1**–**13** at final concentration of 50 µg/mL for 24 h were used to extract total RNA with Trizol Reagent (Invitrogen, USA) as indicated in the protocol. For each sample, 2 μg of total RNA was reverse transcripted using High Capacity cDNA Reverse Transcription Kit with RNAse inhibitor (Applied Biosystem). Following, 50 ng of cDNA from each sample was used in real-time PCR reaction that was performed on an ABI 7300 Real Time PCR System. Gene expression was evaluated using pre-validated Taqman Gene Expression Assays (Applied Biosystems) for NAT1 (Hs00265080_s1), NAT2 (Hs01854954_s1), BAX (Hs00180269_m1), MCL1 (Hs00172036_m1), BCL2 (Hs00153350_m1), CASP3 (Hs00234387_m1), CASP7 (Hs00169152_m1), CASP8 (Hs00154256_m1), CASP9 (Hs00154261_m1), CCNB1 (Hs00259126_m1), CDK1 (Hs00938777_m1), CCNA2 (Hs00153138_m1), CDC20 (Hs00426680_mH) as well as human beta actin (endogenous control). Also, for CYP1A1, CYP2C19, CYP3A4 and GAPDH (endogenous control), forward and reverse primers were used as described by Matei et al. [51]. Results were analyzed with RQ study software (Applied Biosystems) applying ΔΔC_T_ method.

## 4. Conclusions

Starting from isoniazid, six new *N,N*’-diacylhydrazines were obtained, together with three new *N,N,N’*-triacylhydrazines ones; also, the *N,N’*-diacylhydrazines were converted by synthesis in the corresponding new oxadiazoles. All the compounds were characterized by IR, NMR, MS, and elemental analysis. The newly obtained derivatives of isoniazid exhibit different biological activities, depending on their structure. The best antioxidant and antibacterial activities (including anti-*Mycobacterium tuberculosis* effects) were proved by compound **9**. Compounds **7**–**9** determined cell cycle blocking in G0/G1 phase. Moreover, compound **7** proved to be the most toxic, inducing apoptosis in 54% of cells after 72 h, an effect that can be predicted by the increased expression of mRNA *caspase 3* and *7* after a 24 h treatment. The influence of tested compounds on gene expression of some enzymes implicated in drug metabolism indicate that newly synthesized compounds could be metabolized on other pathways than NAT2, spanning adverse effects of isoniazid.

In summary, compound **9** had the best antibacterial activity and could be used as disinfectant agent, and, along with compounds **7** and **8**, seemed to have antitumor potential. Further studies of action mechanism of these compounds on cell cycle may bring some new significant information regarding their activity potential.

## Figures and Tables

**Table 1:** Results obtained in the synthesis of the compounds.

Compound	Yield [%]	m.p. [°C]	*R_f_* ^a,b,c^
INH	-	171–173	0.03 ^a^
**1**	52	128–129	0.50 ^b^
**2**	40	168–169	0.57 ^b^
**3**	25	145–146	0.56 ^b^
**4**	58	173–175	0.17 ^a^
**5**	54	108–111	0.13 ^a^
**6**	33	133–135	0.17 ^a^
**7**	5	Semisolid	0.60 ^a^
**8**	2	Semisolid	0.52 ^a^
**9**	6	Semisolid	0.63 ^a^
**10**	62	210–211	0.72 ^b^
**11**	65	298–299	0.79 ^b^
**12**	68	341–342	0.75 ^b^
**13**	73	249–250	0.60 ^c^

^a^ AcOEt:CH_2_Cl_2_ = 1:1, ^b^ AcOEt:MeOH = 9:1, ^c^ CH_2_Cl_2_:MeOH = 5:1.

**Table 2:** The anti-tubercular evaluation of the new compounds.

Compound	MIC ^a^ [μg/mL]
INH	0.098
**1**	>25
**2**	>25
**3**	>25
**4**	>25
**5**	>25
**6**	25
**7**	25
**8**	25
**9**	6.25
**10**	>25
**11**	>25
**12**	>25
**13**	>25

^a^ Anti-mycobacterial activity (minimum inhibitory concentration (MIC)) against a clinical strain of *M. tuberculosis*.

**Table 3:** The antimicrobial evaluation of the new synthesized compounds.

Compound	INH	1	2	3	4	5	6	7	8	9	10	11	12	13
*Micrococcaceae*														
*Staphylococcus hominis* 1813	−	−	−	−	−	−	−	−	−	+/−	−	−	−	−
*Staphylococcus hominis* 2610/2710	−	−	−	−	−	+/−	+/−	+/−	+/−	+/−	−	−	−	−
*Staphylococcus aureus*	−	−	−	−	−	−	−	+/−	−	+/−	−	−	−	−
*Staphylococcus aureus* 2669	−	−	−	−	−	−	−	+/−	+/−	+	−	−	−	−
*Staphylococcus aureus* 2754	−	−	−	−	−	−	+/−	+/−	−	+	−	−	−	−
*Staphylococcus aureus* ATCC 25923	−	−	−	−	−	−	−	−	−	+	−	−	−	−
*Staphylococcus aureus* ATCC 29213	−	−	−	−	−	+/−	+/−	+/−	+/−	+/−	−	−	−	−
*Coagulase-negative Staphylococcus* sp. 2672	−	−	−	−	−	−	−	−	−	+/−	−	−	−	−
*Coagulase-negative Staphylococcus* sp. 3026	−	−	+/−	−	−	−	−	−	+/−	+/−	−	−	−	−
*Coagulase-negative Staphylococcus* sp. 196	−	−	−	−	−	−	−	+/−	−	+/−	−	−	−	−
*Coagulase-negative Staphylococcus* sp. 1785	−	−	−	−	−	−	−	−	−	+	−	−	−	−
*Streptococcaceae*														
*Enterococcus faecalis* ATCC 29212	−	−	−	−	−	−	−	−	−	+/−	−	−	−	−
*Enterococcus faecalis* 2920	−	−	−	−	−	−	−	−	−	+	−	−	−	−
*Enterobacteriaceae*														
*Enterobacter cloacae* 1845	+	−	−	−	−	−	−	−	−	+/−	−	−	−	−
*Enterobacter cloacae* 3016	−	−	−	−	−	+/−	+/−	+/−	+/−	+	−	−	−	−
*Citrobacter koseri* 1742	−	−	−	−	+/−	+/−	+/−	+/−	+/−	+/−	−	−	−	−
*Morganella morganni* 2810	−	−	−	−	−	−	−	−	−	−	−	−	−	−
*Escherichia coli* 410	−	−	−	−	−	+/−	+/−	−	−	+/−	−	−	−	−
*Escherichia coli* 1455	−	−	−	−	−	+/−	+/−	+/−	+/−	+	−	−	−	−
*Escherichia coli* 1461	−	−	−	−	−	−	+/−	+/−	+/−	+	−	−	−	−
*Escherichia coli* 1777	−	−	−	−	−	+/−	+/−	+/−	−	+/−	−	−	−	−
*Escherichia coli* ATCC 25922	−	−	−	−	−	−	−	+/−	−	+	−	−	−	−
*Klebsiella pneumoniae* 1756	−	−	−	−	−	−	+/−	−	−	−	−	−	−	−
*Klebsiella pneumoniae* 3029	−	−	−	−	−	+/−	+/−	−	−	+/−	−	−	−	−
*Salmonella typhimurium* ATCC 14028	−	−	−	−	−	+/−	+/−	+/−	+/−	+/−	−	−	−	−
*Serratia marcescens* 1142	−	−	−	−	+/−	+/−	+/−	+/−	+/−	+/−	−	−	−	−
*Shigella sonnei* ATCC 25931	−	−	−	−	−	−	−	−	−	−	−	−	−	−
*Pseudomonadaceae*														
*Pseudomonas aeruginosa* ATCC 27853	−	−	−	−	−	−	−	+/−	−	+/−	−	−	−	−
*Pseudomonas aeruginosa* 165	−	−	−	−	−	−	+/−	+/−	+/−	+/−	−	−	−	−
*Pseudomonas aeruginosa* 1144	−	−	−	−	−	+/−	+/−	+/−	+/−	+/−	−	−	−	−
*Pseudomonas aeruginosa* 1150	−	−	−	−	−	+/−	+/−	+/−	−	+/−	−	−	−	−
*Pseudomonas putida* 160	+/−	−	−	−	−	+/−	+/−	+/−	−	+/−	−	−	−	−
*Xanthomonadaceae*														
*Stenotrophomonas maltophilia* 412	−	−	−	−	+/−	+/−	+/−	+/−	+/−	+/−	−	−	−	−
*Moraxellaceae*														
*Acinetobacter baumanii* 122	−	−	−	−	−	−	−	−	−	+	−	−	−	−
*Acinetobacter baumanii* 125	−	−	−	−	−	+/−	+/−	−	−	+	−	−	−	−
*Acinetobacter baumanii* 247	−	−	−	−	−	−	+/−	−	−	+	−	−	−	−
*Acinetobacter baumanii* 411	−	−	−	−	−	+/−	+/−	+/−	+/−	+	−	−	−	−

Susceptible (+); Resistant (−); Intermediate (+/−).

**Table 4:** Apoptosis induction by the new isoniazid derivatives in eukaryotic cells.

Compound	Necrosis [% of Total Cells]	Late Apoptosis [% of Total Cells]	Early Apoptosis [% of Total Cells]	Viable Cells [% of Total Cells]
72 h				
**HT-29**	1.46	0.206	0.057	98.3
**INH**	5.25	3.09	0.915	90.7
**1**	0.854	0.503	0.278	98.4
**2**	0.493	0.937	0.305	98.3
**3**	0.382	0.304	0.294	99.0
**4**	11.1	1.85	2.15	84.9
**5**	12.2	3.28	1.56	83
**6**	5.82	2.7	1.01	90.5
**7**	20.7	33.3	2.93	43
**8**	8.38	7.05	1.99	82.6
**9**	10.8	2.8	1.22	85.2
**10**	0.766	0.317	0.196	98.7
**11**	1.43	0.301	0.457	97.8
**12**	0.553	0.82	0.23	98.4
**13**	0.658	0.239	0.194	98.9
48 h				
**HT-29**	1.27	0.264	0.13	98.3
**INH**	5.96	2.63	0.661	90.8
**1**	2.35	0.325	0.337	97.0
**2**	2.37	0.221	0.431	97.0
**3**	0.706	0.121	0.538	98.6
**4**	4.32	1.5	0.703	93.5
**5**	5.19	4.35	1.17	89.3
**6**	7.28	2.6	0.346	89.8
**7**	16.2	12.4	0.736	70.7
**8**	6.64	2.52	0.614	90.2
**9**	10.1	4.52	0.708	84.6
**10**	0.599	0.359	0.435	98.6
**11**	3.15	0.834	0.337	95.7
**12**	0.734	0.381	0.527	98.4
**13**	0.732	0.174	0.131	99.0
24 h				
**HT-29**	0.68	0.032	0.404	98.9
**INH**	3.68	1.85	1.06	93.4
**1**	0.612	0.411	0.124	98.9
**2**	0.637	0.505	0.204	98.7
**3**	1.1	0.794	0.019	98.1
**4**	5.58	0.964	0.832	92.6
**5**	6.6	0.132	0.237	93
**6**	4.88	1.2	1.14	92.8
**7**	6.51	1.14	0.87	91.5
**8**	4.22	1.71	1.95	92.1
**9**	4.82	1.5	1.32	92.4
**10**	0.835	0.169	0.129	98.9
**11**	1.16	0.127	0.164	98.5
**12**	1.15	0.816	0.11	97.9
**13**	1.4	0.23	0.058	98.3
